# The effects of intranasal oxytocin on the efficacy of psychotherapy for major depressive disorder: a pilot randomized controlled trial

**DOI:** 10.1017/S0033291724000217

**Published:** 2024-07

**Authors:** Mark A. Ellenbogen, Christopher Cardoso, Lisa Serravalle, Kiran Vadaga, Ridha Joober

**Affiliations:** 1 Centre for Research in Human Development, Department of Psychology, Concordia University, Montréal, Canada; 2 The Douglas Research Centre, Department of Psychiatry, McGill University, Montréal, Canada

**Keywords:** intranasal oxytocin, major depressive disorder, psychotherapy, therapeutic alliance

## Abstract

**Background:**

Although both pharmacotherapy and psychological treatments are considered to be efficacious in the treatment of major depressive disorder (MDD), one third of patients do not respond to treatment and many experience residual symptoms post-treatment. In this double-blind placebo-controlled randomized control trial (RCT), we assessed whether intranasal oxytocin (OT) augments the therapeutic efficacy of psychotherapy for MDD and improves the therapeutic alliance.

**Methods:**

Twenty-three volunteers (12 female) with MDD underwent 16 sessions of interpersonal therapy. Prior to each session, volunteers self-administered 24 International Units of intranasal OT (*n* = 12; *Syntocinon*) or placebo (*n* = 11). Depressive symptoms were assessed with the *Inventory of Depressive Symptomatology* at pre- and post-treatment, and at a six month follow-up.

**Results:**

Multilevel modeling found a significant effect of OT on the negative slope of depressive symptoms over time (*p* < 0.05), with medium-large effect sizes at post-treatment (Cohen's *d* = 0.75) and follow-up (Cohen's *d* = 0.82). Drug intervention also predicted the intercept when examining the weekly ratings of the therapeutic alliance (*p* < 0.05), such that volunteers receiving OT, relative to placebo, reported improved therapeutic alliance at session 1. The agreement of goals between therapists and participants, a facet of the therapeutic alliance, mediated the relationship between drug intervention and clinical outcome.

**Conclusion:**

In this pilot study, the administration of intranasal OT, relative to placebo, improved the therapeutic alliance at the beginning of therapy and therapeutic efficacy of psychotherapy in persons with MDD. Future RCTs should attempt to replicate these findings in larger samples with different therapeutic modalities (*ClinicalTrials.gov: NCT02405715*).

Although a range of therapies are considered to be efficacious in the short-term treatment of major depressive disorder (Hollon & Ponniah, 2010; Kennedy et al., 2016; MDD), approximately 40–50% of patients either drop out of treatment prematurely or do not achieve full clinical remission following acute phase treatment with pharmacotherapy (Gitlin, 2014; Rush et al., 2004) or psychological treatments (DeRubeis et al., 2005; Hollon et al., 2005). Many patients with MDD do not maintain their treatment gains following successful treatment, with approximately 30 and 55% of remitted MDD patients evidencing recurrence by one and two years following treatment, respectively (Solomon, 2000; Vittengl, Clark, Dunn, & Jarrett, 2007). Moreover, residual symptoms and poor psychosocial functioning often persist following successful treatment of depressive symptoms, and these are robust predictors of recurrence of MDD (Harkness, Theriault, Stewart, & Bagby, 2014; ten Doesschate, Bockting, Koeter, Schene, & Group, 2010). Given the modest efficacy rates, high recurrence, and persistent residual symptoms among those with MDD, there is an acute need for more efficacious treatment strategies. Various augmentation strategies to improve treatment efficacy, such as adding an additional drug (i.e. antipsychotic, lithium, etc.) or psychological intervention (i.e. mindfulness meditation) have been studied (Dupuy, Ostacher, Huffman, Perlis, & Nierenberg, 2011; Kleeblatt, Betzler, Kilarski, Bschor, & Kohler, 2017; Segal, Williams, & Teasdale, 2002). To date, there has been mixed success with augmentation strategies for the treatment of MDD, and a need for further research (Kleeblatt et al., 2017). In the present study, we propose that exogenous oxytocin (OT) might be useful as a potential augmentation agent in the treatment of MDD.

It is well known that OT is involved in promoting mother–offspring attachment and pair bonding across a variety of animal species through its actions in the central nervous system (Bosch & Young, 2018; Carter, 1998). Human studies of the oxytocinergic system, using the exogenous intranasal administration of OT, indicate a more complex relationship with social behavior. While some studies have found that the administration of intranasal OT, relative to placebo, elicits increases in trust, cooperation, attachment and positive communication (Bernaerts et al., 2017; Ditzen et al., 2009; Kosfeld, Heinrichs, Zak, Fischbacher, & Fehr, 2005; Yang, Wang, Wang, & Wang, 2021), other studies have failed to replicate these findings (Declerck, Boone, Pauwels, Vogt, & Fehr, 2020; Lane et al., 2015) or report opposite effects including increased aggression and gloating in response to competitive games (De Dreu, Greer, Van Kleef, Shalvi, & Handgraaf, 2011; Ne'eman, Perach-Barzilay, Fischer-Shofty, Atias, & Shamay-Tsoory, 2016; Shamay-Tsoory et al., 2009; Zhang, Gross, De Dreu, & Ma, 2019). Contextual factors may explain the heterogeneity observed in the human literature on OT. Based on the theory that OT increases the salience of emotional and social cues, rather than indiscriminately promoting prosocial behavior (Shamay-Tsoory & Abu-Akel, 2016), OT's effects on social behavior might be context-dependent (Bartz, Zaki, Bolger, & Ochsner, 2011; Wong, Cardoso, Orlando, Brown, & Ellenbogen, 2021). In a within-subject placebo-controlled study comparing the effects of OT on perceived emotional support during autobiographical memory recall elicited by a computer (non-social context) or a research assistant (social context), OT *increased* perceived support by the research assistant in the social context among women motivated to affiliate, but *decreased* perceived emotional support in men and women in the non-social context (Cardoso, Valkanas, Serravalle, & Ellenbogen, 2016). Thus, while OT may promote prosocial behavior in contexts where social relationships are available, it may decrease the motivation to affiliate when such relationships are untrustworthy or unavailable. These findings highlight the need to consider context when using of OT therapeutically, particularly in populations with deficient interpersonal functioning such as those with MDD (Joiner & Timmons, 2009).

The use of intranasal OT as a therapeutic agent has been mixed. While studies have reported positive effects in reducing post-traumatic stress disorder symptoms (among those with high acute symptoms at baseline; van Zuiden et al., 2017) and negative symptoms in schizophrenia (Gibson et al., 2014), a number of studies have found that intranasal OT, relative to placebo, failed to decrease symptoms of anxiety (Guastella, Howard, Dadds, Mitchell, & Carson, 2009), autism spectrum disorder (Guastella et al., 2015), and psychotic and negative symptoms in schizophrenia (Buchanan et al., 2021; Cacciotti-Saija et al., 2015; Lee et al., 2013). Little is known about the therapeutic use of OT in persons with MDD (see De Cagna et al., 2019 for review). Although OT had beneficial effects in a case study and one open trial of a small sample of patients with resistant MDD on antidepressant medication (Scantamburlo, Ansseau, Geenen, & Legros, 2011; Scantamburlo, Hansenne, Geenen, Legros, & Ansseau, 2015), neither of the studies included a placebo comparison. A study of 16 patient with postnatal depression (five on OT) found no therapeutic effects of daily OT administration in combination with psychodynamic therapy (Clarici et al., 2015). In this study, OT was administered in the morning and may have been given hours prior to the psychotherapy sessions. Similarly, another study found that a single OT administration, relative to placebo, prior to a 20 min psychotherapy session had no antidepressant effects but increased anxiety in patients with MDD (MacDonald et al., 2013). These results might have occurred because the therapy was too brief and the therapists were instructed to be neutral and unsupportive, both of which might have created a negative context that is atypical of psychotherapy in general (Cardoso & Ellenbogen, 2013). Given the scarcity of data from multi-session randomized controlled trials, it is not known whether the use of adjunct intranasal OT in the treatment of MDD is beneficial. However, there is evidence that individuals with MDD might benefit from the use of OT in the context of psychotherapy. First, the administration of intranasal OT alters social cognition more strongly in persons reporting high sub-clinical depressive symptoms than persons with low depressive symptoms (Boyle, Johnson, & Ellenbogen, 2022; Ellenbogen, Linnen, Cardoso, & Joober, 2013; Ellenbogen, Linnen, Grumet, Cardoso, & Joober, 2012). Thus, depressed individuals may have an increased sensitivity to the administration of intranasal OT. Second, given the context effects described previously (Wong et al., 2021), the positive and supportive nature of a psychotherapy session might be an excellent venue to elicit OT's putative prosocial effects. Moreover, the patient-therapist relationship in psychotherapy, known as the therapeutic alliance, might be a key target of OT's therapeutic potential, given OT's positive effects on interpersonal behaviour among dyads (Ditzen et al., 2009). Indeed, it is well known that the therapeutic alliance is a robust predictor of the efficacy of psychotherapy across a wide range of mental disorders and therapy orientations (Ardito & Rabellino, 2011).

As its primary aim, the present study assessed whether the adjunct administration of OT with psychotherapy, relative to placebo, would improve treatment efficacy in persons diagnosed with MDD. A secondary aim of the study is to assess whether OT improves the participant–therapist relationship, known as the therapeutic alliance, and whether these changes might represent a putative mechanism for the therapeutic effects of OT. Three hypotheses were put forth. First, it was predicted that adjunct intranasal OT, relative to placebo, would lead to lower depression scores at post-treatment and a six-month follow-up. Second, it was predicted that intranasal OT, relative to placebo, would lead to improved participant ratings of the therapeutic alliance during the intervention. Third, it was hypothesized that OT-induced changes in the therapeutic alliance would mediate the relationship between drug administration and treatment efficacy.

## Method

### Participants

**Figure 1.**
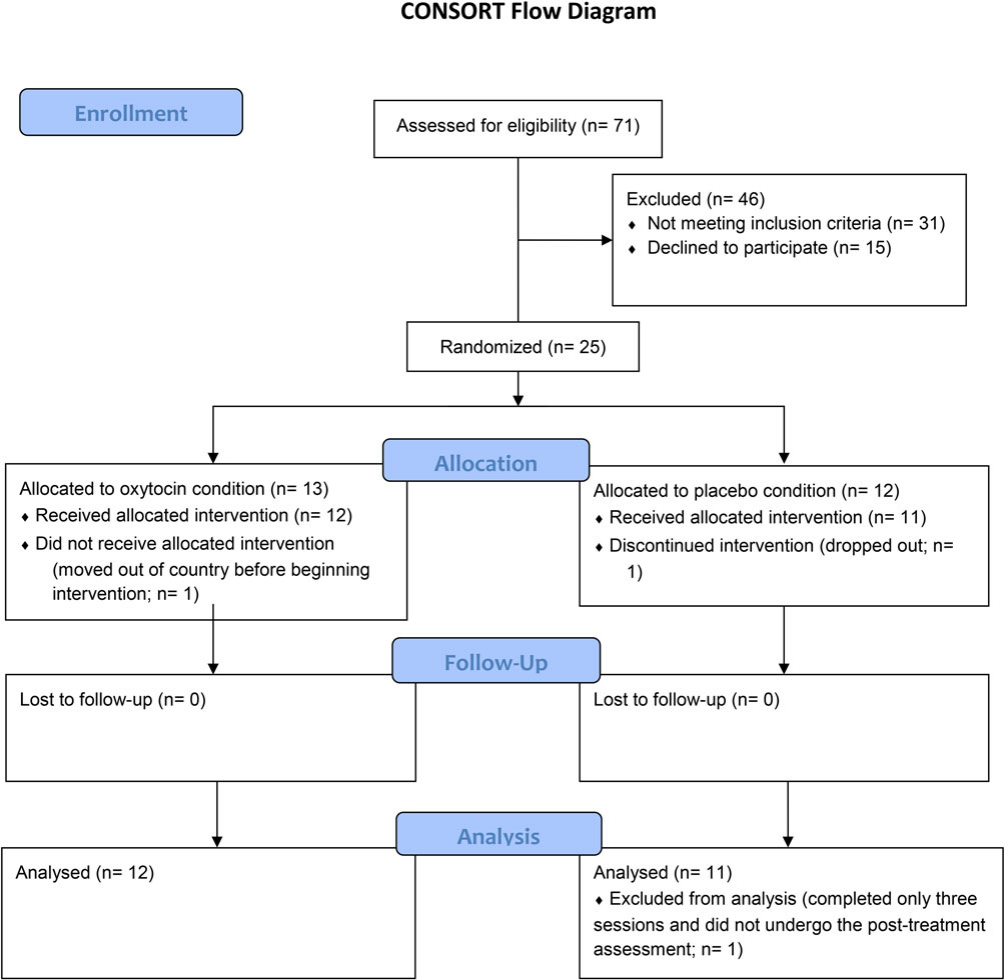
The consort flow diagram describing study recruitment and exclusion into the randomized controlled trial comparing adjunct oxytocin and psychotherapy and placebo and psychotherapy.

Seventy-one English-speaking participants between the ages of 18 and 50 years of age were recruited through advertisements placed online and in print via a free newspaper distributed to subway commuters in Montréal, Canada. Exclusion criteria included (1) major medical illness, in particular, subjects with evidence or history of malignancy or any significant hematological, endocrine, cardiovascular (including any rhythm disorder), respiratory, renal, hepatic, or gastrointestinal disease, (2) current (in the past month) use of any endocrine-relevant or psychotropic medication other than antidepressants, (3) current substance dependence or abuse, (4) use of illicit drugs (stimulants, narcotics, psychedelics/hallucinogens, cannabis, non-prescription medication) in the previous 8 weeks, (5) lifetime history of a psychosis (except if part of MDD) or pervasive developmental disorder, (6) past or current comorbid axis-1 disorder, except dysthymia, adjustment disorder, generalized anxiety disorder, social phobia, and specific phobia, and (7), for females, being pregnant or breastfeeding, or planning to become pregnant. As described in the Consort Flow Diagram (Fig. 1), 25 participants with MDD were randomized into one of two study arms, and 23 participants (12 OT, 11 placebo) completed the intervention and assessments (baseline, post-treatment, and six-month follow-up). Demographic data and comorbid mental disorders are presented in Table 1.

**Table 1.** Baseline and study outcome measures

	Placebo	Oxytocin	
*N*	11	12	
Sex (male/female)	5M/6F	6M/6F	
Mean age (s.d.; range) in years	29.4 (7.5; 21–46)	27.2 (5.4; 20–37)	
Mean (s.d.) education (scale of 1–8^a^)	5.4 (2.2)	4.9 (1.7)	
# (%) Caucasian	9 (81.8)	7 (58.3)	
# (%) on antidepressant medication^b^	1 (9.1)	2 (16.7)	
# (%) with any comorbid diagnosis	5 (45%)	5 (42%)	
# (%) comorbid social phobia	4 (36%)	4 (33%)	
# (%) comorbid generalized anxiety disorder	1(9%)	1(8%)	
# (%) agoraphobia	0	1 (8%)	
# (%) recurrent episode of depression	10 (91)	8 (67)	
Mean (s.d.; range) number of therapy sessions	14.9 (1.6; 11–16)	14.7 (2.1; 9–16)	
Mean (s.d.; range) BDI depression time 1	28.8 (10.9; 8–46)	31.2 (9.9; 14–45)	
Mean (s.d.; range) suicidal behaviour time 1	8.8 (3.7; 4–15)	9.1 (2.8; 3–15)	
Mean (s.d.; range) BAI anxiety time 1	18.7 (8.3; 6–34)	19.3 (13.4; 4–50)	
Mean (s.d.; range) interpersonal stress time 1	10.5 (2.1; 7–14.5)	9.0 (2.8; 4.5–13.5)	
Mean (s.d.; range) non-interpersonal stress time 1	9.4 (2.4; 6.5–13.5)	10.1 (2.5; 6–13)	
Mean (s.d.; range) GAF time 1	59.9 (9.3; 45–80)	57.4 (11.8; 35–75)	
Mean (s.d.; range) social support time 1	4.3 (1.2; 2.6–6.2)	4.1 (1.1; 2.4–5.8)	
Mean (s.d.; range) IDS-C depression time 1	29.4 (6.8; 15–39)	32.8 (11.5; 15–57)	
Mean (s.d.) IDS-C depression time 2	12.2 (8.3)	7.2 (6.6)	
Mean (s.d.) IDS-C depression time 3	10.2 (8.5)	4.7 (4.6)	
Mean (s.d.) ISD-C time 2 minus time 1	−17.2 (9.7)	−25.7 (12.7)	
Mean (s.d.) ISD-C time 3 minus time 1	−19.2 (10.0)	−28.2 (11.8)	
# (%) ⩾ 50% decrease in IDSC scores at time 2	6 (54)	11 (92)	
# (%) ⩾ 50% decrease in IDSC scores at time 3	9 (82)	11 (92)	
Mean (s.d.) WAI-S Patient-Report Alliance Total Session 1	28.4 (7.9)	37.3 (10.7)	
Mean (s.d.) WAI-S Patient-Report Alliance Total Session 8	48.1 (7.6)	50.5 (10.6)	
Mean (s.d.) WAI-S Patient-Report Alliance Total Session 16 (or final session)	51.1 (9.5)	54.6 (6.3)	
Mean (s.d.) WAI-S Goals Subscale Session 1	7.4 (2.5)	10.7 (5.1)	
Mean (s.d.) WAI-S Goals Subscale Session 8	15.9 (2.7)	16.7 (3.6)	
Mean (s.d.) WAI-S Goals Subscale Session 16 (or final session)	16.9 (3.1)	18.3 (2.0)	

s.d., standard deviation; BDI, Beck Depression Inventory; suicidal behaviour from the Suicidal Behavior Questionnaire-Revised; BAI, Beck Anxiety Inventory; interpersonal and non-interpersonal chronic stress are from UCLA Life Stress Interview; GAF, Global Assessment of Functioning; social support from the Multiple Scale of Perceived Social Support; IDS-C, Inventory of Depressive Symptomatology – Clinician Rated; WAIS-S, Working Alliance Inventory-Short Form (patient ratings).

a 1 = Grade 6 or less, 2 = grade 7 to 12 (without graduating high school), 3 = graduated high school or high school equivalent, 4 = part college, 5 = graduated 2 year college, 6 = graduated 4 year college, 7 = part graduate/professional school, 8 = completed graduate/professional school.

b Although not included, one participant in the oxytocin group was taking St. John's wort (300 mg).

*Note*. No significant group differences were found at time 1.

### Materials and measures

#### Structured Clinical Interview for DSM-IV-TR (SCID; First, Spitzer, Gibbon, and Williams, 2002)

The SCID was used to determine participant eligibility into the study. It has strong diagnostic specificity and test–retest reliability (Lobbestael, Leurgans, & Arntz, 2011; Osório et al., 2019). Interviewers rated the Global Assessment of Functioning scale (0–100).

#### Inventory of Depressive Symptomatology – Clinician Rated (IDS-C; Rush et al., 1986)

The IDS-C is a 30 item structured interview for assessing the severity of DSM-IV symptoms of MDD, with high internal consistency (Cronbach's alpha, *α* = 0.94), strong criterion validity, and excellent sensitivity to treatment effects (Rush et al., 1986; Rush, Gullion, Basco, Jarrett, & Trivedi, 1996; Trivedi et al., 2004). Participants also underwent a second structured interview, the Hamilton Depression Rating Scale (HAM-D), and completed the Beck Depression Inventory (Beck, Steer, & Brown, 1996) and Beck Anxiety Inventory (Beck & Steer, 1993).

#### Working alliance inventory – short form, patient, and therapist version (WAI-S; Horvath and Greenberg, 1989)

The WAI-S assesses three key aspects of the therapeutic alliance: agreement of goals (outcomes) of therapy (4-items), agreement on tasks of therapy (4-items), and the development of a bond between the patient and therapist (4-items). The WAI-S demonstrates strong internal consistency (patient: *α* = 0.93; therapist: *α* = 0.87) and criterion validity (Horvath & Greenberg, 1989). Internal consistency in the present sample for the patient version were 0.89, 0.82, 0.80, and 0.75 for the total score, goals, tasks, and bond subscales of the WAI-S.

#### Other measures

To assess group differences at baseline, study participants completed the UCLA Life Stress Interview (Hammen, Shih, Altman, & Brennan, 2003), Suicidal Behavior Questionnaire- Revised (Osman et al., 2001), Beck Anxiety Inventory (Beck & Steer, 1993), and the Multiple Scale of Perceived Social Support (Canty-Mitchell & Zimet, 2000).

### Procedure

Following a telephone screening, eligible participants were invited for a laboratory visit, where they provided written informed consent, completed a battery of questionnaires and underwent a diagnostic assessment using the SCID, as well as an assessment of clinician-rated depressive symptoms using the IDS-C and the Hamilton Depression Rating Scale. Senior graduate students in clinical psychology, who received extensive training in administering the SCID and IDS-C, conducted the interviews. If participants were eligible for the study, a visit to a private health clinic for a routine physical examination and blood work, and a serum pregnancy test for women, were scheduled. Next, participants were randomized into one of two treatment arms: Interpersonal Psychotherapy (IPT) with adjunct intranasal OT or IPT with adjunct placebo. Treatment allocation was based on a computer-generated randomization sequence using block-randomization with a ratio of 1:1 and block sizes of four. Allocation concealment with respect to drug condition was achieved by using pre-determined envelope-concealed assignment, administered by a laboratory coordinator not involved in the assessment of potential participants. Importantly, therapists and participants were blind to treatment allocation, as the nasal sprays were identical with respect to appearance, taste, smell, and administration procedure.

Participants underwent up to 16 50-minute sessions of IPT conducted by four (2 male; 2 female) senior graduate students in clinical psychology, trained in IPT through accredited workshops. The principal investigator (Dr Ellenbogen), also trained in IPT, supervised the therapy sessions through weekly meetings with therapists. IPT is a time-limited empirically supported psychological treatment of MDD that focuses on ameliorating interpersonal difficulties most closely related to the depressive episode (Weissman, Markowitz, & Klerman, 2000). Thirty minutes prior to each session, participants self-administered 24 I.U. of intranasal OT (*Syntocinon*, Novartis) or a placebo with matched inactive ingredients, under the supervision of the therapist. Drug administration was conducted in accordance with published guidelines on intranasal OT administration (Guastella et al., 2013). Following each session, participants and therapists completed ratings of the therapeutic alliance (WAIS-S). Participants underwent assessments of their depressive symptoms, chronic stress (not reported, except at time 1), personality (not reported), and social functioning (not reported, except at time 1) at baseline, at the end of the therapy, and at a six-month follow-up. Measures collected in the study but not reported in the present manuscript are reported in online Supplemental Table S1. Senior graduate students in clinical psychology who were blind to the treatment allocation conducted the assessments. Participants were remunerated $120 CAD for their participation at each assessment phase. The project was approved by the Human Research Ethics Committee at Concordia University (Montreal, Canada) and was registered at *ClinicalTrials.gov* (registration number: NCT02405715).

### Statistical analyses

Growth-curve multilevel modelling using Hierarchical Linear Modeling (version 8.0; Raudenbush, Bryk, Cheong, Congdon, & du Toit, 2019) was used to assess these data. Multilevel modeling has distinct advantages with data such as these because it can accommodate for violations of the statistical assumption of independence in sampling. A person's depression scores at a given time point is inherently dependent on the previous depression score and will subsequently influence later time points. At level 1 (within-subject), we estimated the variance in depression scores across the three phases of testing as a function of the uncentered scores of time and a residual term. The coefficient of primary interest was the estimation of the slope (time), which examined changes in depression across time. Since we did not expect differences at baseline because the study design was a randomized controlled trial, we constrained the intercept to be fixed for the level 2 model. This would allow for all of the potential between-subject variability to be associated with differences in the changes over time, which is the central aim of the study. At level 2 (between-subject), intervention group and control measures (sex of the participant and education as a proxy of socioeconomic status) were used to account for variability observed in the level-1 slope. The interaction between group and sex of the participant was assessed but was subsequently dropped from the analyses because it did not add anything to the model. All level 2 predictor variables were standardized prior to conducting the analyses. The analyses of therapeutic alliance were conducted in the same fashion, except that both slope and intercept were modelled at level 2. Intercept for therapeutic alliance was of interest because it denotes therapeutic alliance at the end of the first session. Only linear effects for changes in depression and therapeutic alliance scores across time are presented. Modeling with quadratic trends over time did not add anything new to the model.

The reported effects are based on models using restricted maximum likelihood estimation and robust standard errors. Chi-square and logistic regression were used to examine whether the drug intervention improved response rates, defined as a 50% decline in IDSC scores from baseline. Exploratory analyses using ordinary least squares regression with 95% confidence interval bias-corrected bootstrapping (Hayes, 2018) were conducted to assess whether changes in the therapeutic alliance mediated the relationship between the intervention group and change in IDS-C depression scores.

## Results

Means, standard deviations, and frequencies of baseline and outcome variables, as well as the number of sessions completed, are presented in Table 1. No baseline group differences were observed.

### The effect of adjunct intranasal OT on depression scores across time

**Figure 2.**
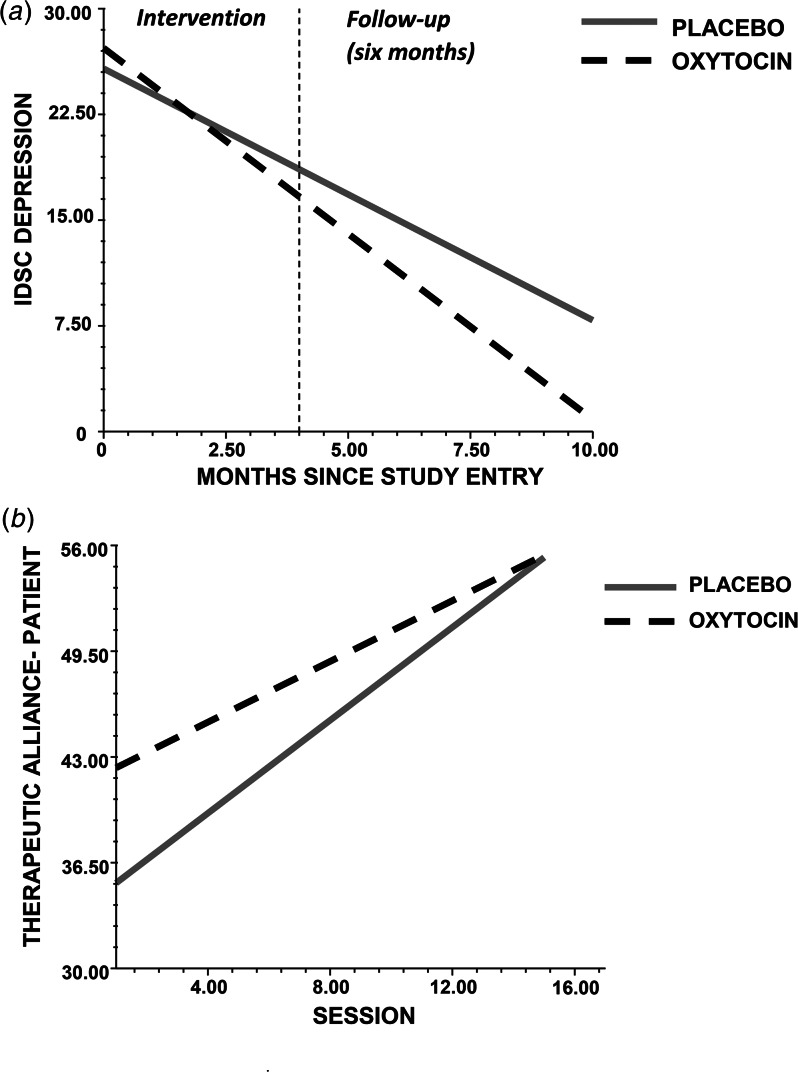
a. The administration of intranasal oxytocin, relative to placebo, increased the rate of improvement (slope) on the Inventory of Depressive Symptomatology-Clinician Rated (IDS-C) at post-treatment and at a six-month follow-up in persons with major depressive disorder undergoing interpersonal psychotherapy. b. The administration of intranasal oxytocin, relative to placebo, improved participants' ratings of the therapeutic alliance on the Working Alliance Inventory-Short Form at session 1 (intercept) in persons with major depressive disorder. The rate of improvement (slope) of the placebo group across 16 sessions of interpersonal therapy was greater than that of the oxytocin group.

Multilevel modelling analyses, presented in Table 2 (top), were conducted to estimate the effect of intervention group (adjunct OT *v.* placebo) on IDS-C scores across the three time points (pre-, post-intervention, follow-up; Table 1). The level 1 model for IDS-C scores at baseline (intercept) and change over time (slope) found significant effects for the intercept and slope, indicating that participants' IDS-C scores at baseline (*p* < 0.001) and across time (*p* < 0.001) were significantly different from zero. Next, the effect of intervention group was added to the level 2 model, along with sex and education as covariates. Group was a significant predictor of slope (*p* < 0.05), such that patients receiving OT exhibited a steeper slope in IDS-C scores over time (Fig. 2, panel A). Relative to the level 1 model with only time entered, the addition of the intervention group led to a 33% decrease in between-subject variability in slope. A likelihood ratio test indicated that the level 2 random effects model with predictors provided a better fit of the data than the null or unconditional fixed effect model, χ^2^ (2) = 52.4, *p* < 0.001.

**Table 2.** Effects of intervention group (oxytocin versus placebo) on clinician ratings of depressive symptoms (top) across time (baseline; post-intervention; six-month follow-up) and patient-report therapeutic alliance (bottom) across 16 sessions

	IDSC depression			
	Intercept (baseline/time 1)		Slope (time)	
	Coefficient (s.e.)	*T* ratio	Coefficient (s.e.) *T* ratio	
Level 1 (*b*_0_; *b*_1_)^a^	26.5 (1.5)	17.2**	−2.2 (0.2)	−9.9**
Level 2: Main effects				
Intercept	26.5 (1.5)	17.5**	−2.2 (0.2)	−10.7**
Sex	−0.30 (1.5)	−0.2	0.01 (0.20)	0.06
Education	0.49 (1.3)	0.4	−0.06 (0.15)	−0.42
Intervention group	0.75 (1.5)	0.5	−0.43 (0.21)	−2.12*
	WAI-S therapeutic alliance			
	Intercept (baseline/session 1)		Slope (time)	
	Coefficient (s.e.)	*T* Ratio	Coefficient (s.e.) *T* ratio	
Level 1 (*b*_0_; *b*_1_)^a^	37.7 (1.9)	22 4***	1.2 (0.1)	11.7***
Level 2: Main effects				
Intercept	37.6 (1.7)	22.4***	1.18 (0.08)	−14.8***
Sex	0.43 (1.9)	0.23	0.02 (0.08)	0.26
Education	2.24 (2.2)	1.02	−0.17 (0.08)	−2.05
Intervention group	3.87 (1.8)	2.19*	−0.25 (0.08)	−3.13**

*Notes*. IDSC, Inventory of Depressive Symptomatology, Clinician Rating; s.e., standard error; WAI-S, Working Alliance Inventory-Short Form (patient report).

a The first parameter estimated (*b*_0_) estimated the intercept, which represents participants depressive symptoms at time 1 and therapeutic alliance at session 1, and the second parameter (*b*_1_) estimates the slope, which represents the within-person change over time in depressive symptoms and across session for the therapeutic alliance.

*p < 0.05; **p < 0.01; ***p < 0.001.

Effect sizes (Cohen's *d*) were computed by comparing IDS-C change scores (T2 minus T1 and T3 minus T1; see Table 1) between groups. Effects sizes for the intervention were 0.75, 95% CI (−0.10 to 1.59), and 0.82, 95% CI (−0.033 to 1.67), at post-intervention and follow-up, respectively, which are considered to be in the medium to large range (Cohen, 1988). The intervention had a significant effect on the number of participants who achieved a 50% decline in symptoms at Time 2 (response rate) from their baseline IDS-C score (χ^2^[1, *N* = 23] = 4.1, *p* = 0.043), but not at Time 3 (χ^2^ [1, *N* = 23] = 0.49, *p* = 0.48; see Table 1). The absence of an intervention effect on the response rate at Time 3 was due to a ceiling effect at Time 2, as 11/12 patients receiving adjunct OT had already achieved clinical response at Time 2.

The same analyses were conducted on HAM-D scores. In the multilevel analyses, intervention group also predicted steeper slope in HAM-D scores over time, but this effect fell short of conventional levels of statistical significance (*p* = 0.062; online Supplemental Tables S2 and S3). Effects sizes for the intervention were 0.49, 95% CI (−0.34 to 1.32), and 0.76, 95% CI (−0.09 to 1.61), at post-intervention and follow-up, respectively, which are considered to be in the medium to large range (Cohen, 1988). Similar results were found for the Beck Depression Inventory (online Supplemental Tables S2 and S3), with medium effect sizes of 0.54, 95% CI (−0.32 to 1.35), and 0.40, 95% CI (−0.43 to 1.22), at post-intervention and follow-up respectively. There were no effects of group on Beck Anxiety Inventory scores across time (online supplemental Tables S2 and S3).

### The effect of adjunct intranasal OT on the therapist–patient relationship across time

Multilevel modelling analyses, presented in Table 2 (bottom), estimated the effect of intervention group on patient-reported therapeutic alliance scores across 16 sessions. The Level 1 model for therapeutic alliance scores at baseline (intercept) and change over time (slope) found significant effects for the intercept and slope, indicating that participants' ratings of therapeutic alliance at baseline (*p* < 0.001) and across time (*p* < 0.001) were significantly different from zero. Next, the effect of intervention group was added to the level 2 model, along with sex and education as covariates. Intervention group was a significant predictor of the intercept (*p* < 0.05), such that patients receiving OT, relative to placebo, reported higher therapeutic alliance scores at the beginning of therapy, after session 1 (Fig. 2, panel B). Relative to the level 1 model with only time entered, the addition of the intervention group led to an 11.6% decrease in between-subject variability in intercept. Intervention group was also a significant predictor of slope (*p* < 0.01). However, for this effect, participants *receiving placebo* showed a steeper slope than patients receiving OT across the 16 sessions, indicating that patients in the placebo group improved their therapeutic alliance over time to catch up to the gains of the OT group early in therapy (Fig. 2, panel B). Relative to the level 1 model with only time entered, the addition of the intervention group led to a 30.2% decrease in between-subject variability in slope. A likelihood ratio test indicated that the level 2 random effects model with predictors provided a better fit of the data than the null or unconditional fixed effect model, χ^2^ (2) = 270.7, *p* < 0.001.

Effect sizes (Cohen's d) were computed by comparing therapeutic alliance scores at session one and across the first four sessions between groups. Effects sizes were 0.89, 95% CI (0.06–1.73), and 0.86, 95% CI (0.22–1.69) respectively, which are considered to be large effect sizes (Cohen, 1988). No effect of intervention group was found for the therapist-rated therapeutic alliance (see online Supplemental Tables S2 and S3).

### Do changes in the therapist–patient relationship early in therapy mediate the relationship between the intervention and depression scores post-intervention?

Mediation analyses tested whether the drug intervention indirectly reduced Time 2 IDS-C depression (T2 minus T1) through therapeutic alliance scores at session 1, based on the robust intercept (session 1) finding from the previous section. The bias-corrected bootstrap 95% confidence interval for the indirect effect based on 10 000 bootstrap samples found no evidence of mediation (Confidence intervals [CI] were not entirely above or below zero, which is the measure of statistical significance in this analysis), *ab* (indirect effect) = −3.35, s.e. = 3.24; CI −11.9 to 0.53. The indirect effect, *ab* = −3.90, s.e. = 2.95, CI −11.2 to 0.07, for the mediation at time 3 (T3 minus T1) also failed to show evidence of significant mediation, although here it fell just short of statistical significance.

**Figure 3.**
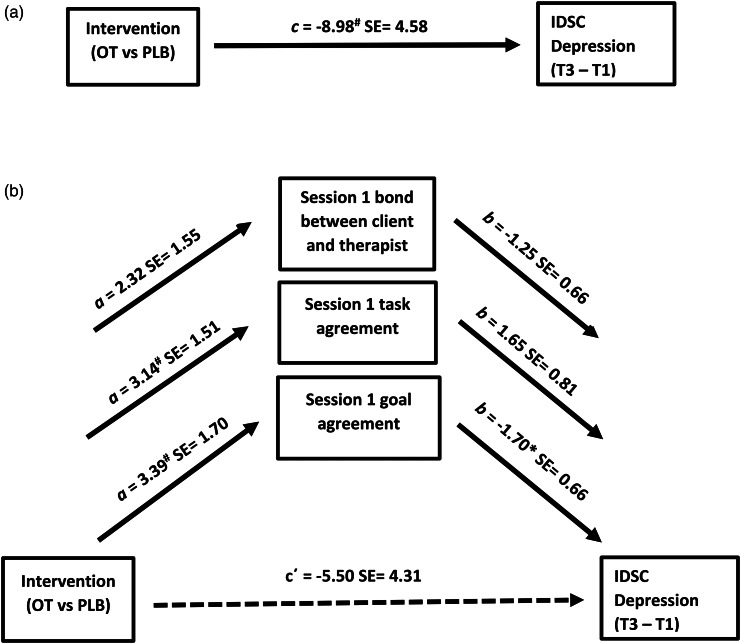
Parallel mediation model testing the agreement of goals, agreement of tasks, and bond between patients and therapist scales of the Working Alliance Inventory- Short Form (patient report) at session 1 as a mediator of the relationship between intervention group (oxytocin, OT, *v.* placebo, PLB) and time 3 (T3) change from baseline (T1) on the Inventory of Depressive Symptomatology, Clinician Rating (IDSC). A. Path *c* is the total effect of intervention group on change in depression scores (sum of direct and indirect effects; *c* = *c΄* + *ab*). B. Path *c΄* is the direct effect of intervention group on change in depression, path *a* is the direct effect of intervention group on therapeutic alliance, and path *b* is the direct effect of therapeutic alliance on change in depression. The indirect effect (*ab*) of intervention group predicting change in depression through the mediators found that the goal agreement scale was significant (confidence intervals: −15.6 to −0.03). Coefficients are unstandardized regression coefficients. ^#^: *p* < 0.065 + ; *p < 0.05.

We then conducted a parallel mediation assessing whether any of the three sub-scales of the WAI-S (bonding, agreement of tasks, and agreement of goals) mediated the relationship between drug intervention and time 3 IDS-C depression. For the agreement of goals sub-scale (see Fig. 3), the bias-corrected bootstrap 95% confidence interval for the indirect effect, *ab* = −5.75, s.e. = 4.05, based on 10 000 bootstrap samples was entirely below zero (−15.6 to −0.03), indicating significant mediation. Mediations with the bonding and tasks sub-scales were not statistically significant at time 3, and no mediations for the three scales were significant at time 2 (data not shown). In sum, improved therapeutic alliance goal agreement mediated the relationship between adjunct OT administration and improvements in IDS-C depression scores at time 3.

## Discussion

Consistent with the primary hypothesis, persons with MDD who underwent up to 16 sessions of psychotherapy with adjunct intranasal OT showed a greater reduction of depressive symptoms at post-treatment and a six-month follow-up than those receiving psychotherapy with adjunct placebo administration. Consistent in part with our second hypothesis that intranasal OT would improve the therapeutic alliance during psychotherapy, we found that OT improved the patient-reported therapeutic alliance at the beginning of therapy relative to placebo, an effect that disappeared over time. Finally, as the third hypothesis, we predicted that OT-induced changes in the therapeutic alliance would mediate the relationship between drug administration and treatment efficacy. Although the hypothesis was not supported with the full score of the therapeutic alliance measure, OT-induced changes in the subscale assessing the agreement of therapeutic goals between therapists and patients mediated the relationship between drug administration and therapeutic efficacy at the six-month follow-up.

OT improved the treatment of MDD when administered in the context of an empirically supported psychotherapy with trained therapists. Although previous studies have found limited support of the efficacy of OT as a therapeutic agent for MDD (De Cagna et al., 2019), the literature is scarce and the few studies examining the effects of OT on MDD symptoms have been methodologically weak (Clarici et al., 2015; MacDonald et al., 2013). Intranasal OT reduced symptoms of depression and post-traumatic stress disorder (PTSD), and improved the therapeutic alliance, during exposure therapy in 17 patients with PTSD, but none of these effects were statistically significant (Flanagan, Sippel, Wahlquist, Moran-Santa Maria, & Back, 2018). The use of OT in individual psychotherapy has advantages over other types of treatment in that it can control for proximal contextual factors that might hinder OT effects when, for example, the drug is self-administered by patients at home in the context of their poor relationships and other negative environmental factors (Guastella et al., 2015). There is growing evidence that OT's effects on human behavior are context-dependent, in that OT administered in non-optimal conditions (during competition, alone with no social contact, etc.) can elicit negative effects (Alcorn, Green, Schmitz, & Lane, 2015; Shamay-Tsoory & Abu-Akel, 2016; Wong et al., 2021). Group therapy, compared to individual psychotherapy, might not be as effective in harnessing OT's therapeutic effects and has yielded mixed results. Among males with methamphetamine use disorder, intranasal OT administered prior to six motivational interviewing group therapy sessions elicited higher attendance to sessions than those who received placebo (Stauffer et al., 2020), but did not alter outcome measures of their addiction. Studies of schizophrenia using OT combined with group social cognition and/or social skills training found no effects of OT, relative to placebo, on clinical outcome measures (Cacciotti-Saija et al., 2015; Davis et al., 2014; Strauss et al., 2019). Thus, it may be the intimacy and closeness of a dyadic therapeutic relationship in multi-session psychotherapy, as shown by OT's improvements in the early therapeutic alliance in the present study, which are critical for OT's therapeutic benefits.

The pattern of findings in the present study suggests that OT is eliciting its therapeutic benefits by enhancing participants' perception of aspects of the therapeutic alliance in the beginning of therapy. This effect was strongest, and statistically significant, only for the agreement of therapeutic goals between therapists and participants. This aspect of the therapeutic alliance is a critical step in the early stages of therapy and has been shown to predict positive therapeutic outcomes more strongly than ratings of the therapeutic bond (Khalifian, Beard, Björgvinsson, & Webb, 2019; Webb et al., 2011). OT's effects on the therapeutic alliance in the present study are consistent with research showing that OT in saliva and plasma are positively associated with positive therapeutic outcomes in MDD (Jobst et al., 2018; Zilcha-Mano, Goldstein, Dolev-Amit, & Shamay-Tsoory, 2021), possibly being driven by changes in the therapeutic alliance (Zilcha-Mano, Shamay-Tsoory, Dolev-Amit, Zagoory-Sharon, & Feldman, 2020). Thus, studies of naturalistic OT levels during psychotherapy for MDD provide converging evidence that changes in the therapeutic alliance might be central in OT's effects on improving psychotherapy outcomes in persons with MDD.

The results of the present study, particularly those related to the therapeutic alliance, are consistent with the general view that intranasal OT administration facilitates trust and cooperative behavior when administered in a social context that provides appropriate outlets for such behavior (Ditzen et al., 2009; Kosfeld et al., 2005; Van IJzendoorn & Bakermans-Kranenburg, 2012; Yang et al., 2021), although there is still controversy over the replicability of these findings (Declerck et al., 2020; Walum, Waldman, & Young, 2016). One limitation of this literature is the lack of studies examining the effects of repeated OT administrations over time. In a study of two weeks of daily OT or placebo administrations in 40 men, OT reduced attachment avoidance and increased attachment toward peers compared to placebo, with the strongest effects being in those persons reporting high insecure attachment to peers at baseline (Bernaerts et al., 2017). These findings are congruent with the results of the present study, where participants received up to 16 weekly intranasal OT administrations. Similarly, persons with MDD might be more sensitive to OT and its contextual effects than populations with no history of MDD (Boyle et al., 2022; Ellenbogen et al., 2013). Participants with high depressive symptoms, for example, were more sensitive to a manipulation of context than those with low depressive symptoms, in that across two studies depressed participants reported more negative autobiographical memories following OT relative to placebo when the task was administered by computer (no social contact) than when administered by an attentive research assistant (Wong et al., 2021). Possibly, this might explain the stronger findings observed here in persons with MDD than in other clinical populations (Guastella et al., 2009; Stauffer et al., 2020). Unfortunately, there are still few OT studies of persons with MDD and fewer of individual psychotherapy to draw strong conclusions.

There are several study limitations. First, the sample size was small and certain analyses (mediation) were underpowered. However, the present study benefitted from the repeated measures design (data from three assessments and up to 16 sessions). Moreover, the sample size in the present study was larger than others in the literature assessing OT in persons with MDD (Clarici et al., 2015; Jobst et al., 2018; MacDonald et al., 2013) and similar to other studies assessing novel therapeutics such psilocybin-assisted therapy (*n* = 24; Davis et al., 2021). Second, the present findings are limited to a community sample of persons with mild to moderate MDD, which may not generalize to inpatient samples and persons with severe MDD. Third, the present findings are limited to the use of interpersonal therapy in the treatment of MDD. It is not known whether they can be extended to more common psychological treatments of MDD such as cognitive-behavioral therapy. Fourth, important non-psychiatric outcomes such as quality of life were not directly assessed in the study. In sum, the present study demonstrated that repeated intranasal administrations of OT prior to psychotherapy sessions, compared to placebo, improved therapeutic outcomes at post-treatment and a six-month follow-up in persons with MDD. The therapeutic effects of OT appear to be driven by the early improvement of the therapeutic alliance at the beginning of therapy, particularly on the agreement of goals between therapists and participants. Future research in this area will need to replicate these findings in larger samples and using different empirically supported psychological interventions.

## Supporting information

Ellenbogen et al. supplementary material 1 Ellenbogen et al. supplementary material

Ellenbogen et al. supplementary material 2 Ellenbogen et al. supplementary material

Ellenbogen et al. supplementary material 3 Ellenbogen et al. supplementary material

## Supplementary material

The supplementary material for this article can be found at https://doi.org/10.1017/S0033291724000217
