# Supplementary material for: The effects of intranasal oxytocin on the efficacy of psychotherapy for major depressive disorder: a pilot randomized controlled trial
Source: Psychol Med. 2024 Mar 6;54(9):2122–32. doi: 10.1017/S0033291724000217 (PMC11413360; doi:10.1017/S0033291724000217)
Supplement: Ellenbogen et al. supplementary material 1 — Ellenbogen et al. supplementary material [file S0033291724000217sup001.pdf]

Supplemental Table 1. Measures collected in the sample but not reported in the manuscript

	Collected at each assessment	Collected after each therapy session
Columbia Suicide Severity Rating Scale (Posner et al, 2011	X	
UCLA Life Stress Interview (Hammen et al, 2003)[1]	X	
Multiple Scale of Perceived Social Support (Canty-Mitchell & Zimet, 2000)[1]	X	X
Beck Anxiety Inventory (Beck et al, 1988)[2]		X
Beck Depression Inventory II (Beck et al, 1996)[2]		X
Social Adjustment Scale – Self-report Short Form (Gameroff et al, 2012)	X	X
Toronto Alexithymia Scale (Bagby et al, 1994).	X	
Suicidal Behavior Questionnaire- Revised (Osman et al., 2001)[1]	X	
Ruminative Response Style Questionnaire (Nolen-Hoeksema & Morrow, 1991)	X	
Experiences in Close Relationships (Fairchild & Finney, 2006).	X	
Adult Attachment Scale (Collins & Read, 1990).	X	
Perceived Stress Scale (Cohen et al, 1983)	X	X
NEO Personality Inventory – Revised (Costa & McCrae, 1992).	X	
Helpful Aspects of Therapy (Elliott, 2010)		X
Modified Autobiographical Memory Task (Williams & Broadbent, 1986).	X	
Diurnal salivary cortisol levels (across two days, 6 samples per day	X	

Note. [1] reported at time 1 in the manuscript, but the measure was collected all phaese of the study. [2] reported at all study phases in the manuscript, but not those assessed after each therapy sessions
