# Supplementary material for: The effects of intranasal oxytocin on the efficacy of psychotherapy for major depressive disorder: a pilot randomized controlled trial
Source: Psychol Med. 2024 Mar 6;54(9):2122–32. doi: 10.1017/S0033291724000217 (PMC11413360; doi:10.1017/S0033291724000217)
Supplement: Ellenbogen et al. supplementary material 2 — Ellenbogen et al. supplementary material [file S0033291724000217sup002.pdf]

Supplemental Table 2. Mean HAM-D, BDI, BAI, and WAI-S scores (SD)

	Placebo	Oxytocin
HAM-D Depression Time 1 (+ range)	29.4 (10.9; 8 - 46)	32.8 (9.9; 14 - 45)
HAM-D Depression Time 2	12.2 (8.3)	7.2 (6.6)
HAM-D Depression Time 3	10.2 (8.5)	4.7 (4.6)
HAM-D Time 2 minus 1	-9.3 (3.6)	-12.5 (8.5)
HAM-D Time 3 minus 1	-8.9 (5.3)	-13.9 (7.6)
BDI Depression Time 1 (+ range)	28.8 (10.9; 8 - 46)	31.2 (9.9; 14 - 45)
BDI Depression Time 2	12.7 (10.5)	9.1 (7.5)
BDI Depression Time 3	11.4 (10.1)	8.4 (14.6)
BDI Time 2 minus 1	-16.1 (12.1)	-22.2 (11.5)
BDI Time 3 minus 1	-17.4 (10.7)	-22.8 (16.0)
BAI Anxiety Time 1 (+ range)	18.7 (8.3; 6 - 34)	19.3 (13.4; 4 - 50)
BAI Anxiety Time 2	6.0 (6.2)	6.58 (6.7)
BAI Anxiety Time 3	6.4 (3.4)	7.7 (10.1)
BAI Time 2 minus 1	-12.7 (6.3)	-12.7 (12.6)
BAI Time 3 minus 1	-12.4 (9.1)	-11.6 (17.0)
Mean WAI-S Therapist-Report Alliance Total Session 1	30.8 (7.9)	35.2 (8.7)
Mean WAI-S Therapist-Report Alliance Total Session 8	41.5 (7.8)	44.6 (42.5)
Mean WAI-S Therapist-ReportAlliance Total Session 16 (or final session)	44.6 (4.6)	46.7 (3.8)

Note. HAM-D: Hamilton Depression Rating Scale; BDI: Beck Depression Inventory; BAI: Beck Anxiety Inventory; WAI-S: Working Alliance Inventory-Short Form
