# Supplementary material for: The effects of intranasal oxytocin on the efficacy of psychotherapy for major depressive disorder: a pilot randomized controlled trial
Source: Psychol Med. 2024 Mar 6;54(9):2122–32. doi: 10.1017/S0033291724000217 (PMC11413360; doi:10.1017/S0033291724000217)
Supplement: Ellenbogen et al. supplementary material 3 — Ellenbogen et al. supplementary material [file S0033291724000217sup003.pdf]

Supplemental Table 3. Effects of intervention group (oxytocin versus placebo) on clinician ratings of depressive symptoms (HAM-D) and therapeutic alliance (WAI-S), and self-report of depressive (BDI) and anxiety symptoms (BAI) across time (baseline; post-intervention; six-month follow-up)

	**HAM-D Depression**			
	Intercept (Time 1)		Slope (Time)	
	Coefficient (SE)	T-Ratio	Coefficient (SE)	T-Ratio
**Level 1 (b_0; b_1)[a]**	12.7 (0.88)	14.4***	-1.07 (0.13)	-7.98***
**Level 2: Main effects**				
Intercept	12.6 (0.85)	15.0***	-1.06 (0.13)	-8.47***
Sex	0.61 (0.86)	0.71	-0.06 (0.11)	-0.53
Education	-0.22 (0.64)	-0.34	0.01 (0.08)	0.10
Intervention Group	0.78 (0.87)	0.89	-0.24 (0.12)	-1.98[#]

	**BDI Depression**			
	Intercept (Time 1)		Slope (Time)	
	Coefficient (SE)	T-Ratio	Coefficient (SE)	T-Ratio
**Level 1 (b_0; b_1)[a]**	25.8 (1.67)	15.5***	-1.89 (0.28)	-6.84***
**Level 2: Main effects**				
Intercept	25.7 (1.64)	15.7***	-1.84 (0.28)	-6.61***
Sex	-0.63 (1.52)	-0.42	-0.25 (0.34)	-0.73
Education	0.78 (2.01)	0.39	0.00 (0.37)	0.01
Intervention Group	0.45 (1.80)	0.25	-0.28 (0.25)	-1.12

	**BAI Anxiety**			
	Intercept (Time 1)		Slope (Time)	
	Coefficient (SE)	T-Ratio	Coefficient (SE)	T-Ratio
**Level 1 (b_0; b_1)[a]**	22.8 (3.3)	6.9***	-6.00 (1.38)	-4.34***
**Level 2: Main effects**				
Intercept	22.7 (3.20)	7.10***	-5.95(1.31)	-4.53***
Sex	1.88 (3.23)	0.58	-1.16 (1.44)	-0.81
Education	-1.50 (2.47)	-0.61	1.37 (1.29)	1.06
Intervention Group	-0.06 (3.35)	-0.02	0.31 (1.38)	0.23

	**WAI-S therapist-rated therapeutic alliance**			
	Intercept (Time 1)		Slope (Time)	
	Coefficient (SE)	T-Ratio	Coefficient (SE)	T-Ratio
**Level 1 (b_0; b_1)[a]**	36.7 (0.97)	38.1***	0.68 (0.07)	10.5***
**Level 2: Main effects**				
Intercept	36.8 (0.75)	48.9***	0.68 (0.06)	10.5***
Sex	-2.35 (0.85)	-2.77*	0.23 (0.08)	3.03**
Education	-0.72 (0.87)	-0.83	-0.05 (0.07)	-0.73
Intervention Group	0.98 (0.77)	1.27	-0.08(0.07)	-1.28

BAI: Beck Anxiety Inventory; WAI-S: Working Alliance Inventory-Short Form

[a] The first parameter (b_0) estimated the intercept, which represents participants' depressive symptoms at time 1, and the second parameter (b_1) estimates the slope, which represents the within-person change over time in depressive symptoms.
